# Design, Co-Expression, and Evaluation for Assembly of the Structural Proteins from Thermophilic Bacteriophage ΦIN93

**DOI:** 10.3390/ijms26115201

**Published:** 2025-05-28

**Authors:** Hong Liu, Milad Kheirvari, Ebenezer Tumban

**Affiliations:** Graduate Program in One Health Sciences, School of Veterinary Medicine, Texas Tech University, Amarillo, TX 79106, USA; hong.liu@ttu.edu (H.L.); milad.kheirvari@ttu.edu (M.K.)

**Keywords:** thermophilic bacteriophage ΦIN93, protein co-expression, thermophilic bacteria, BL21 Star, membrane-associated proteins, coat proteins

## Abstract

Bacteriophage ΦIN93 has an icosahedral-like capsid that is believed to be composed of two putative capsid or coat proteins, namely open reading frame (ORF)13 and ORF14. In addition to the two capsid proteins, there are other proteins that may be associated with the structure of the virus. For example, five other proteins (ORF12, ORF16, ORF17, ORF19, and ORF20) in the virus have been identified as putative membrane-associated proteins. It is believed that membrane-associated proteins associate with coat proteins (serve as scaffolding proteins) to promote viral assembly. While the expression/co-expression of ORF13 and ORF14 have been done to assess if they can assemble to form virus-like particles (VLPs), the expression of any of the membrane-associated proteins and their contribution to assembly have never been attempted. In this study, we successfully co-expressed, for the first time, three membrane-associated proteins (ORF12, ORF16, ORF17) in addition to ORF13 and ORF14 in thermophilic bacteria (*Thermus thermophilus*, *HB27:nar* strain) and in mesophilic bacteria (BL21 Star). The expression levels of the proteins were higher in BL21 Star than in *Thermus thermophilus*, *HB27:nar*. Some of the expressed proteins (especially ORF17) migrated at sizes that were more than their deduced molecular weight (based on amino acid sequence). Co-expression of these proteins did not lead to the formation of structures that we believe are VLPs. Nevertheless, we believe co-expressing these proteins together from different plasmids is a good approach to assess which of them may be required to form VLPs.

## 1. Introduction

Phi IN93 (ΦIN93) is a thermophilic virus that was isolated (in 1995) from a thermophilic bacterium, *Thermus aquaticus,* in a hot spring in Japan; *Thermus aquaticus* has an optimum growth temperature of 75 °C [1]. The genome of the virus, double-stranded circular DNA (~19.6 kbp), is surrounded by a polyhedral (icosahedral-like) capsid that is composed of two putative coat proteins [open reading frame (ORF)13 and ORF14] [2,3]. ORF13 and ORF14 are considered capsid proteins due to their sequence homology to the small major capsid protein (VP16) and large major capsid protein (VP17), respectively, of thermophilic bacteriophage P23-77. ORF13 and ORF14 are ~80% and ~73% identical (in amino acids) to VP16 and VP17 of P23–77, respectively (Table 1) [2,3]. The three-dimensional (3D) structure of ORF13 and ORF14 have been predicted, using in silico approach, based on a model structure of VP16 and VP17 [4]. ORF13 (amino acids 21–165) forms a homodimer, in the middle, that interacts with two copies of ORF14 (amino acids 46–271; one on the left and the other on the right).

In a previous study, we co-expressed and purified (for the first time) from *E. coli*, ORF13 and ORF14 with the ultimate goal of assessing whether the expressed coat proteins could form virus-like particles (VLPs) [4]. Transmission electron microscopy (TEM) analysis of co-expressed ORF13 and ORF14 showed oval structures that were not VLPs; the structures were less than the size (130 nm) of an authentic ΦIN93 virus [1]. VLPs are empty shells derived from the expression of structural proteins. Over-expression of these structural proteins in a suitable host allows the proteins to spontaneously self-assemble into VLPs. Proteins on VLPs are arranged geometrically into dense, repetitive manner. Thus, VLPs are morphologically/antigenically similar to viruses, except that they do not contain the viral genome.

VLPs have diverse applications in biomedical sciences; they have been used for vaccine design, delivery of cargo to cells, as standards in assays, etc. [5,6]. VLPs derived from thermophilic viruses (if successfully developed) may have several advantages compared to current VLP platforms derived from mesophilic bacteria: (i) they may be thermostable at high temperatures; for example, the capsid of thermophilic phage, P23-77, is 6 nm thick and its coat proteins are stable at >80 °C [3,7,8]. Thus, VLPs derived from these proteins may be thermostable at high temperatures; (ii) thermophilic viruses infect thermophilic bacteria that do not colonize humans nor animals. Thus, humans and animals lack pre-existing antibodies against these viruses compared to viruses that colonize humans and animals; as such, VLPs derived from thermophilic viruses (if used for vaccine design) will be highly immunogenic due to lack of pre-existing antibodies compared to VLPs derived from human/animal viruses. Pre-existing antibodies to some vaccine platforms reduce the immunogenicity of the platforms as well as efficacy of heterologous antigens displayed on the platforms [1,9,10,11,12,13,14]; and (iii) the diameter of ΦIN93 and P23-77 is 130 nm and 78 nm, respectively [1,8], suggesting that VLPs derived from these thermophilic bacteriophages may have a bigger diameter compared to other VLPs; a bigger diameter implies that the VLPs can be loaded with more vaccine adjuvants, imaging fluorophores, or cargo for targeted delivery to cells.

To build on our previous work (which assessed if co-expression of ORF13 and ORF14 could form VLPs), we explored here whether the addition of more viral structural proteins to ORF13 and ORF14 expression would lead to the formation of VLPs. Based on studies with the P23-77 bacteriophage [3], the capsid of ΦIN93 may be composed/associated with other structural proteins (membrane-associated proteins) in addition to ORF13 and ORF14. Membrane-associated proteins are part of an internal lipid membrane, which is believed to lie underneath the capsid of P23-77. Five proteins (ORF12, ORF16, ORF17, ORF19, and ORF20) in ΦIN93 have been identified as putative membrane-associated proteins based on amino acid identity with proteins on bacteriophage P23–77 (Table 1) [3]. Although the role of these membrane-associated proteins in ΦIN93 virus are not clear, it is believed that the association of membrane-associated proteins (in some viruses) with coat proteins promotes viral assembly, i.e., membrane-associated proteins serve as scaffolding proteins during assembly (reviewed in [15]). Thus, we decided to explore here if the co-expression of these membrane-associated proteins together with ORF13 and ORF14 will promote assembly of the proteins into VLPs. In this study, we successfully co-expressed, for the first time, three membrane-associated proteins (ORF12, ORF16, ORF17) in addition to ORF13 and ORF14 in two types of bacteria: we co-expressed them in a closely related natural host of the virus (*Thermus thermophilus* bacterium, *HB27:nar* strain) and in BL21 Star(DE3) strain of *E. coli*. *Thermus thermophilus* is a close relative *to Thermus aquaticus*, and it is susceptible to ΦIN93 [16]. The goal of expressing these proteins in a thermophilic bacterium was to take advantage of features (temperature, ionic strength, and pH) that have been shown to be important for the survival of thermophages, including their assembly. Temperature, ionic strength, and pH affect assembly of coat proteins to VLPs, as well as their stability [17,18,19,20,21]. *Thermus thermophilus HB27:nar* is a derivative of HB27 thermophilic bacteria that carries a respiratory nitrate reductase (nar) operon; the operon allows the bacteria to grow both aerobically and anaerobically [22].

## 2. Results

### 2.1. The DNA of the Coat Proteins and Membrane-Associated Proteins (Non-Codon Optimized) Were Successfully Amplified by PCR in the Presence of 3% Dimethyl Sulfoxide (DMSO)

**Figure 1:**
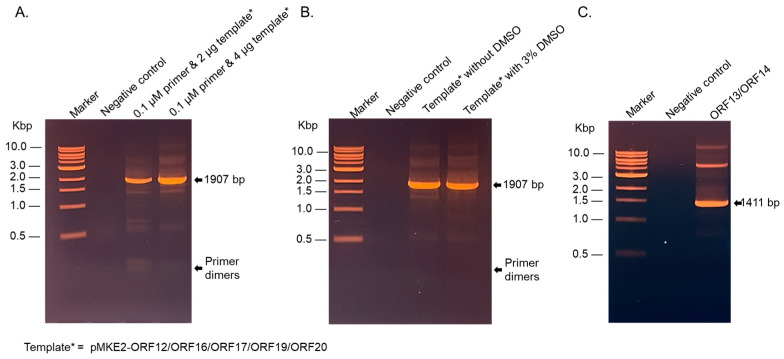
Amplification of ORF fragments. (**A**) Agarose gel of PCR reaction mixtures using different concentration of primer pair and pMKE2-ORF12/ORF16/ORF17/ORF19/ORF20 as template. PCR conditions were as follows: Initial denaturation, 94 °C for 2 min, denaturation 94 °C for 30 s, annealing 63 °C for 30 s, extension 68 °C for 2 min, final extension 70 °C for 5 min. (**B**) PCR reactions in (**A**) in the presence of DMSO with annealing temperature of 65 °C. (**C**) Agarose gel of PCR amplification of ORF13/ORF14 using PCR conditions in (**B**). Distilled water was used instead of template as negative controls for PCRs in (**A**–**C**).

The cloning of ORF13/ORF14 and ORF12/ORF16/ORF17/ORF19/ORF20 to the thermophilic vector (pMKE2) was performed by PCR amplification followed by restriction digestion and ligation to digested pMKE2. The GC content of ORF13/ORF14 and that of ORF12/ORF16/ORF17/ORF19/ORF20 is 65.5–66%. This made it very challenging to amplify the fragments by PCR. The GC content of the forward and reverse primers (sections that bind to the template) to amplify ORF13/ORF14 were 61.9% and 61.1%, respectively. Those for the forward and reverse primers (sections that bind to the template) to amplify ORF12/ORF16/ORF17/ORF19/ORF20 were 81% and 50%, respectively. Attempts to amplify the gene fragments (using 0.2 µM of primer and 1 µg of template) were unsuccessful with the following PCR conditions: initial denature temperature (94 °C for 2 min), denature temperature (94 °C for 30 s)*, annealing temperature (60 °C for 30 s)*, extension temperature (68 °C for 2 min)*, and final extension temperature (70 °C for 5 min). PCR conditions in asterisks were performed using 30 cycles prior to the final extension step. Only a faint band of ~1411 base pairs (expected size for ORF13/ORF14 fragment) in addition to multiple nonspecific smeary bands were observed with ORF13/ORF14 primer pair. For ORF12/ORF16/ORF17/ORF19/ORF20 amplification, only a strong band of primer dimers was observed. PCRs were repeated with the same reaction mixture and conditions, but with annealing temperatures of 55 °C and 58 °C; only primer dimers were visible on agarose gel. Reductions in PCR primers to 0.1 µM and increases in the template (ORF12/ORF16/ORF17/ORF19/ORF20) to 2 µg and 4 µg at an annealing temperature of 63 °C gave rise to a DNA band of 1907 bp (as expected for the fragment); the band from the PCR reaction using 4 µg template was twice that from 2 µg template (Figure 1A). However, both PCR reactions still had faint smeary non-specific bands. To reduce non-specific bands and to increase the amplified template, 3% DMSO was added to the PCR mixture (with 0.1 µM primer and 4 µg of template) and PCR was repeated at the same conditions, but with an annealing temperature of 65 °C [23]. This enhanced the PCR reaction; a strong band of 1907 bp was observed (Figure 1B). These conditions/reaction mixtures were used to amplify ORF13/ORF14 for cloning to pMKE2 plasmid (Figure 1C).

### 2.2. ORF13/ORF14 Coat Proteins and ORF12/ORF17 Membrane-Associated Proteins Were Successfully Expressed in T. thermophilus HB27:nar but at Different Levels

**Figure 2:**
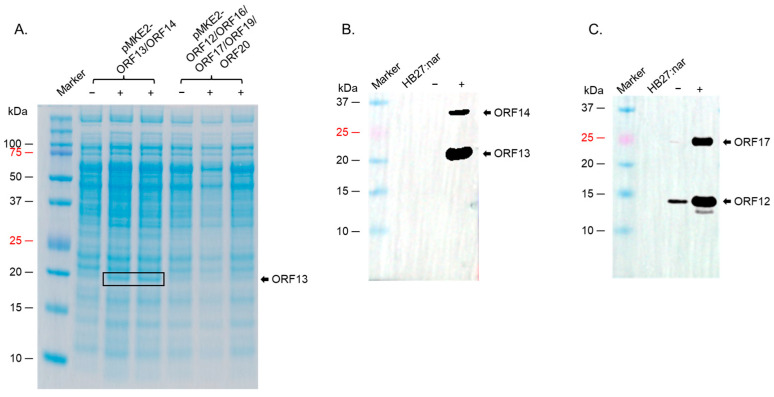
SDS PAGE of the expression of ORF13/ORF14 or ORF12/ORF16/ORF17/ORF19/ORF20 in *T. thermophilus HB27:nar.* (**A**) Bacterial lysates (from co-expressed ORF13/ORF14 culture or co-expressed ORF12/ORF16/ORF17/ORF19/ORF20 culture induced with 40 mM KNO_3_ for 4 h) were separated on SDS PAGE gel and stained with Coomassie Blue. (**B**) Lysates from (**A**) were run on SDS PAGE gel and Western blot conducted using a mixture of anti-ORF13 and anti-ORF14 polyclonal sera (at 1:1000 and 1:500 dilutions, respectively). (**C**) Lysates from (**A**) were run on SDS PAGE gel and Western blot conducted with anti-ORF12-ORF16-ORF17-ORF19-ORF20 polyclonal sera (1:1500 dilution). HB27:nar: bacteria without pMKE2 vector, (−): uninduced culture, (+): induced culture.

To assess the expression of the individual proteins, *T. thermophilus HB27:nar* was transformed with the expression plasmids pMKE2-ORF13/ORF14 or pMKE2-ORF12/ORF16/ORF17/ORF19/ORF20, and protein expression was induced as described below. For pMKE2-ORF13/ORF14 construct, only ORF13 (19.3 KDa) was expressed as evident by SDS PAGE gel (Figure 2A). For the pMKE2-ORF12/ORF16/ORF17/ORF19/ORF20 construct, no expression was evident for any of the proteins by SDS PAGE (Figure 2A). To assess whether ORF14 (~32.0 KDa) from the pMKE2-ORF13/ORF14 construct, or ORF12, ORF16, ORF17, ORF19, ORF20 from pMKE2-ORF12/ORF16/ORF17/ORF19/ORF20 were also expressed, but at low levels, we performed Western blots with cell lysates from bacteria (transformed with the plasmids) using: (i) mixed polyclonal sera from mice immunized separately with ORF13 and ORF14 as primary antibodies or (ii) polyclonal sera from mice immunized with the recombinant protein (ORF12-ORF16-ORF17-ORF19-ORF20) as primary antibodies. As shown in Figure 2B, ORF14 was also expressed, but at low levels. For ORF12/ORF16/ORF17/ORF19/ORF20, a band of ~14.6 KDa, which corresponds to the expected size of ORF12, was observed in induced samples (Figure 2C); however, a small band of the same size as ORF12 was observed in uninduced lysates but not in the lysates from bacteria without the vector. In addition to the ~14.6 KDa band for ORF12, a band around ~25 KDa was also observed (Figure 2C); we believe this band is ORF17, even though the size is bigger than the deduced molecular weight of ORF17 (23.3 KDa, based on amino acid sequence). ORF19 and ORF20 were not expressed.

**Figure 3:**
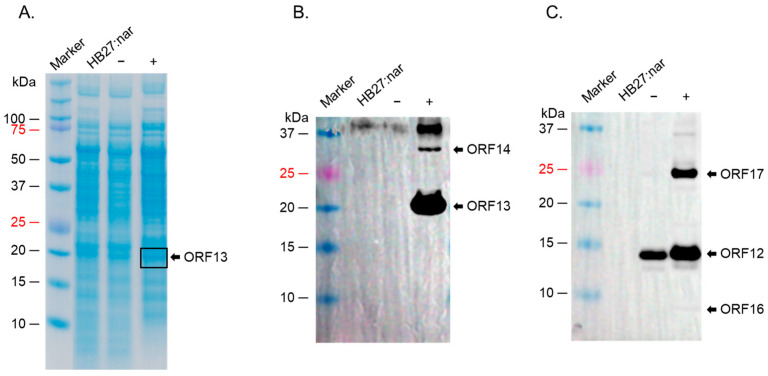
SDS PAGE of co-expression of ORF13/ORF14 and ORF12/ORF16/ORF17/ORF19/ORF20 in *T. thermophilus HB27:nar.* (**A**) Bacterial lysates (from co-expressed ORF13/ORF14 and ORF12/ORF16/ORF17/ORF19/ORF20 cultures induced with 40 mM KNO_3_ for 4 h) were separated on SDS PAGE gel and stained with Coomassie Blue. (**B**) Lysates from (**A**) were run on SDS PAGE gel and Western blot conducted using a mixture of anti-ORF13 and anti-ORF14 polyclonal sera (1:1000 and 1:500, respectively). (**C**) Lysates from (**A**) were run on SDS PAGE gel and Western blot conducted with anti-ORF12-ORF16-ORF17-ORF19-ORF20 polyclonal sera (1:1500 dilution). HB27:nar: bacteria without pMKE2 vector, (−): uninduced culture, (+): induced culture.

Given the expression of some of these proteins, we decided to see if we could co-express the capsid proteins (pMKE2-ORF13/ORF14 construct) with membrane-associated proteins (pMKE2-ORF12/ORF16/ORF17/ORF19/ORF20 construct), with the long-term goal of assessing whether co-expressing these proteins may lead to the formation of VLPs. Of all seven proteins, only ORF13 could be seen on SDS PAGE gel (Figure 3A). Analyses of lysates of the co-expressed constructs (by Western blot) confirmed the expression of ORF13 (Figure 3B); ORF14 was expressed at low levels. In addition to these two proteins, ORF12 and ORF17 (~14.6 KDa and ~25 KDa bands, respectively) were also expressed (Figure 3C). A faint band below 10 KDa was also observed in induced samples. This may be ORF16 (which is 8.9 KDa). As with previous experiments above, a small band of the same size as ORF12 was observed in uninduced lysates but not in the lysates from bacteria without the vector. ORF19 and ORF20 were not expressed (same as in Figure 2).

**Figure 4:**
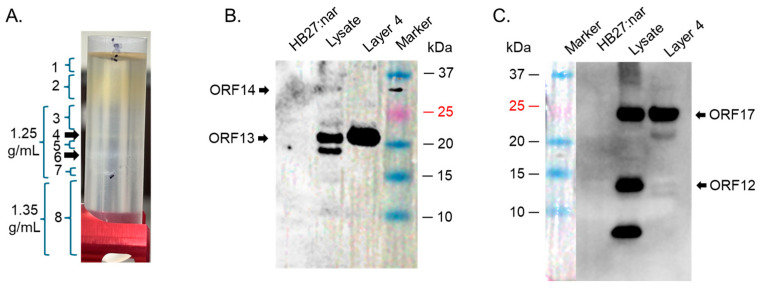
Cesium chloride gradient ultracentrifugation of ORFs expressed in HB27:nar. (**A**) HB27:nar culture expressing ORFs was lysed with Bugbuster buffer and the supernatant was put on 1.25 g/mL and 1.35 g/mL cesium chloride density gradient. The tubes were spun as described in the text and layers (numbered 1-8) analyzed for coat proteins. Layer 4 from (**A**) was used to do Western blot using: (**B**) a mixture of anti-ORF13 and ORF14 antibodies (at 1:2000 and 1:250, respectively), and (**C**) anti-ORF12-ORF16-ORF17-ORF19-ORF20 recombinant protein polyclonal antibodies (at 1:1500 dilution).

To assess whether the proteins can form VLPs, supernatants expressing the proteins were run on a cesium chloride gradient. The density gradient of bacteriophage ΦIN93 has not yet be determined. Thus, we did ultracentrifugation at different density gradients starting from 1.14 g/mL to 1.65 g/mL and analyzed different factions (layers 1–8) for coat proteins and/or membrane-associated proteins by Coomassie blue and Western blots. Most of the viral proteins were detected using 1.25 and 1.35 g/mL density gradients in layer 4 (Figure 4A). As shown in Figure 4B,C, ORF13 and ORF17 was detected in this layer by Western blot. In addition to that, a faint band around ~14.6 KDa was also evident (bands could only been seen with high gamma level with Azure 600 image system). TEM analysis of this layer showed oval structures with an average size of ~62 nm (Supplementary Materials, Figure S1A).

### 2.3. ORF12, ORF13, ORF14, ORF16, and ORF17 Were Successfully Co-Expressed in a Strain of E. coli (BL21 Star)

**Figure 5:**
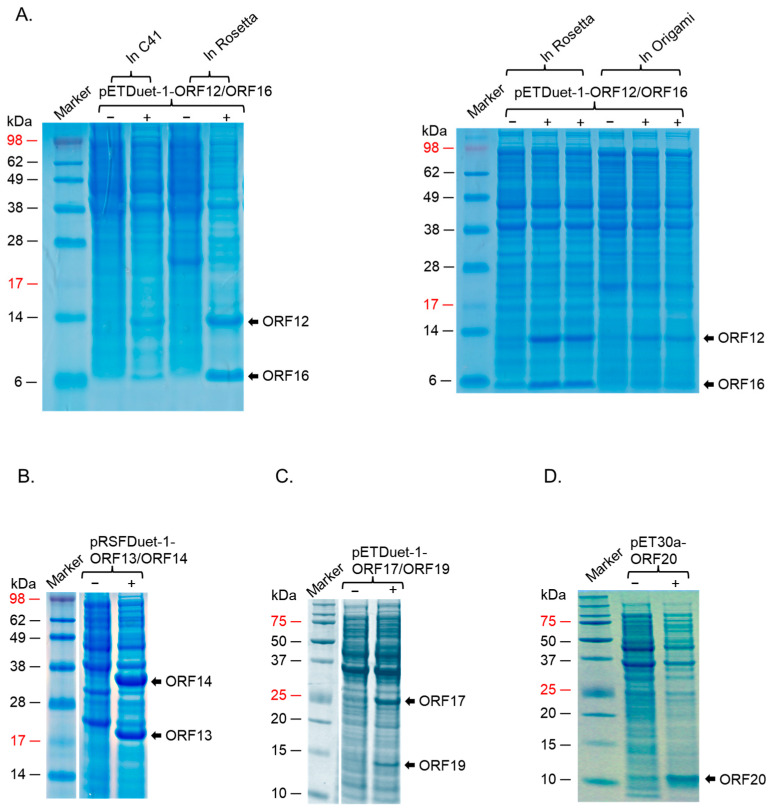
SDS PAGE of the expression of coat proteins and membrane-associated proteins in different *E. coli* strains. (**A**) C41, Rosetta 2, and Origami 2 cells were transformed with equal concentration of pETDuet-1 co-expressing ORF12 and ORF16. (**B**) Rosetta 2 cells were transformed with pRSFDuet-1 co-expressing ORF13 and ORF14. (**C**) Rosetta 2 cells were transformed with pETDuet-1 co-expressing ORF17 and ORF19. (**D**) BL21 star cells were transformed with pET30a expressing ORF20-his tag. In all cases, protein expression was induced and assessed as described in the text. (−): uninduced culture, (+): induced culture.

**Figure 6:**
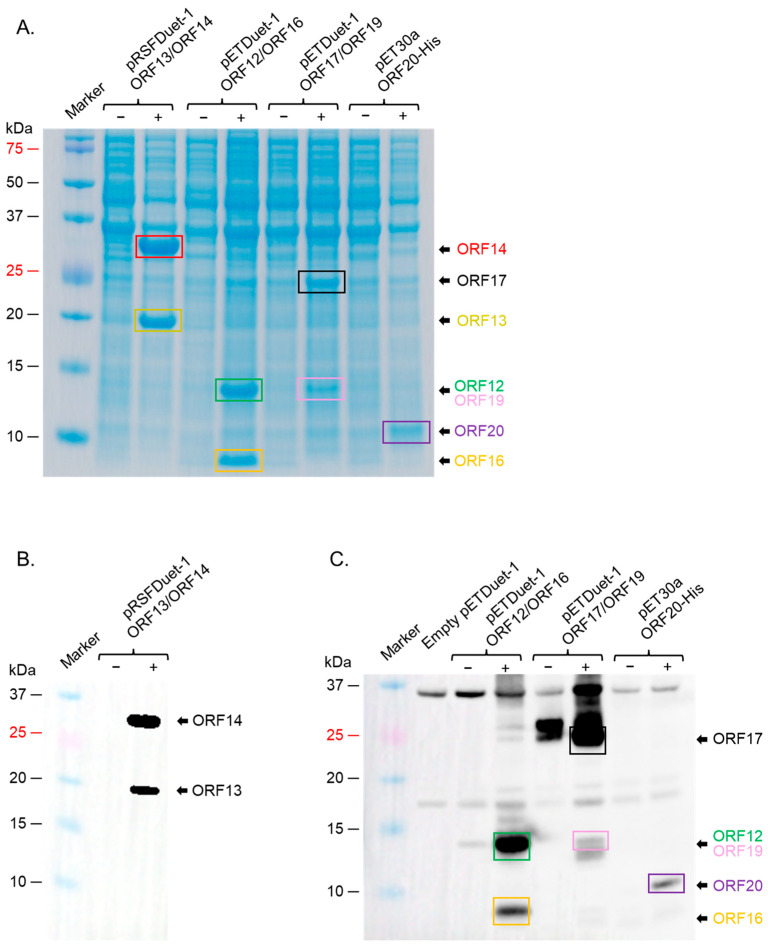
SDS PAGE of the expression of coat proteins and membrane-associated proteins in BL21 star; (**A**) BL21 star cells were transformed with indicated plasmids. Protein expression was induced and assessed as described in the text. Lysates from samples in (**A**) were run on SDS PAGE and Western blot conducted using a mixture of anti-ORF13 (1:1000) and anti-ORF14 sera (1:500) (**B**) or anti-ORF12-ORF16-ORF17-ORF19-ORF20 polyclonal sera (1:1500) (**C**). (−): uninduced culture, (+): induced culture.

**Figure 7:**
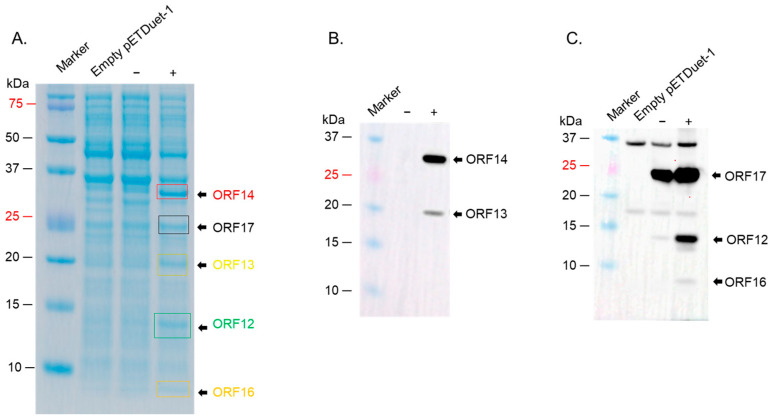
SDS PAGE of the co-expression of coat proteins and membrane-associated proteins in BL21 star; (**A**) BL21 star cells were transformed with a mixture of 4 plasmids. Protein expression was induced and assessed as described in the text. Lysates from samples in (**A**) were run on SDS PAGE and Western blot conducted using a mixture of anti-ORF13 (1:1000) and anti-ORF14 sera (1:500) (**B**) or anti-ORF12-ORF16-ORF17-ORF19-ORF20 polyclonal sera (1:1500) (**C**). (−): uninduced culture, (+): induced culture.

For expression in *E. coli*, we first decided to see which strains of the bacteria will express the proteins at high levels. We first assessed the co-expression of ORF12 and ORF16 cloned in pETDuet-1 vector in three *E. coli* expression systems: C41, Rosetta 2, and Origami 2 cells; while the proteins could be co-expression in all three strains, expression levels in Rosetta 2 cells were the best (Figure 5A, left and right panels). ORF13 and ORF14 cloned in either pETDuet-1 or pRSFDuet-1 vectors could also be co-expressed at high levels in this strain (Figure 5B). ORF17 and ORF19 in pRSFDuet-1 vector and ORF20 in pCDFDuet-1 vector were not expressed in any of the bacterial strains. To assess if these proteins could be expressed using the pET vectors, given the expression of ORF12 and ORF16 pETDuet-1, ORF17 and ORF19 were cloned into pETDuet-1 (ORF17 in MCSI and ORF19 in MCSII) and ORF20 in pET30a; the antibiotic-resistance gene in pETDuet-1-ORF17/ORF19 vector was changed from ampicillin to streptomycin to enable co-expression of the vectors downstream. As shown in Figure 5C, ORF17 and ORF19 could be co-expressed using the pET vector in Rosetta 2 cells; however, ORF20 could not be expressed. Analysis of ORF20 sequence showed that the protein has a transmembrane region (15 amino acids). Removal of the transmembrane region (and the addition of six histidine tag for purification, a TEV cleavage site, and seven amino acids, for removal of the tag) led to the expression of the protein at low levels in Rosetta 2 cells. To assess if the protein could be expressed at high levels in other *E. coli* strains, we transformed the plasmid to BL21 Star cells. As shown in Figure 5D, ORF20 was expressed at high levels in these cells. Given the fact that ORF20 could only be expressed at high levels in BL21 Star cells, and given the fact that we had plan to co-express all seven proteins in the same bacteria to assess assembly of the proteins to VLPs, we decided to see if the other proteins above could be expressed (in pairs) in BL21 Star. As shown in Figure 6A, ORF13 and ORF14 was expressed using pRSFDuet-1 in BL21 Star, ORF12 and ORF16 using pETDuet-1 vector, ORF17 and ORF19 using pETDuet-1 vector and ORF20 using pET30a vector in BL21 Star. The expression of these proteins was confirmed by Western blots (Figure 6B,C). To assess whether co-expression of all these proteins in BL21 Star could lead to assembly, all four expression vectors were transformed into the bacteria. While the expression of ORF12, ORF13, ORF14, ORF16, and ORF17 (to an extent; a faint band) could be visualized on SDS PAGE, the expressions of ORF19 and ORF20 could not be visualized (Figure 7A). To confirm these results, we did Western blots. As shown in Figure 7B,C, Western blot only confirmed the expression of ORF12, ORF13, ORF14, ORF16, and ORF17.

**Figure 8:**
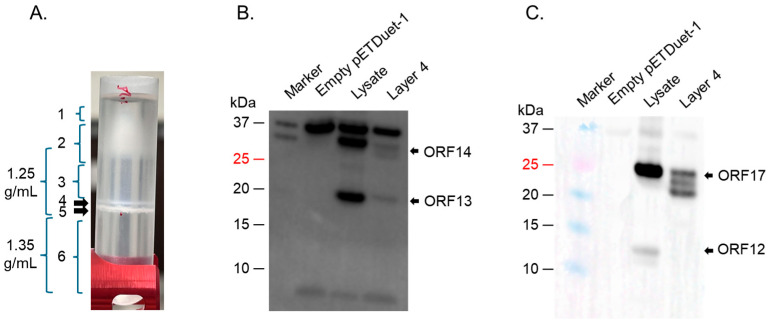
Cesium chloride gradient ultracentrifugation of ORFs expressed in BL21 star cells. (**A**) BL21 star culture expressing ORFs was lysed with Bugbuster buffer and the supernatant was put on 1.25 g/mL and 1.35 g/mL cesium chloride density gradient. The tubes were spun as described in the text and layers (numbered 1–6) analyzed for coat proteins. Layer 4 from (**A**) was used to do Western blot using: (**B**) a mixture of anti-ORF13 and ORF14 antibodies (at 1:1000 and 1:500, respectively), and (**C**) anti-ORF12-ORF16-ORF17-ORF19-ORF20 recombinant protein polyclonal antibodies (at 1:1500 dilution).

To assess whether the proteins can form VLPs, supernatants expressing the proteins were run on a cesium chloride gradient. As shown in Figure 8, only faint bands corresponding to ORF13, ORF14, and ORF17 could be observed (Western blot) on samples from layer 4. TEM analysis of this layer showed oval structures with an average size of ~100 nm (Supplementary Materials, Figure S1B).

## 3. Discussion

**Figure 9:**
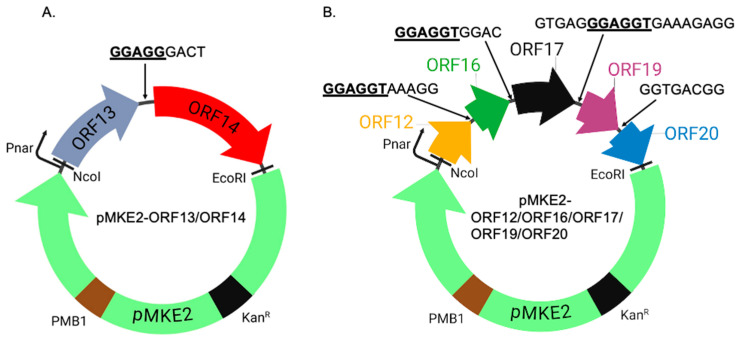
Design of the expression of ORFs in *HB27:nar* bacteria. (**A**) Fragment ORF13 and ORF14 was cloned into pMKE2 plasmid using NcoI and EcoRI sites. (**B**) Fragment ORF12, ORF16, ORF17, ORF19, and ORF20 were cloned into pMKE2 plasmid using the same restriction sites. Linker sequences between ORFs as they appear in the genome are shown in capital letters. Native Shine–Dalgarno sequence in ΦIN93 genome between ORFs are bolded and underlined. Pnar: nitrate reductase promoter. PMB1: Origin of replication. Kan^R^: kanamycin antibiotic resistance gene.

**Figure 10:**
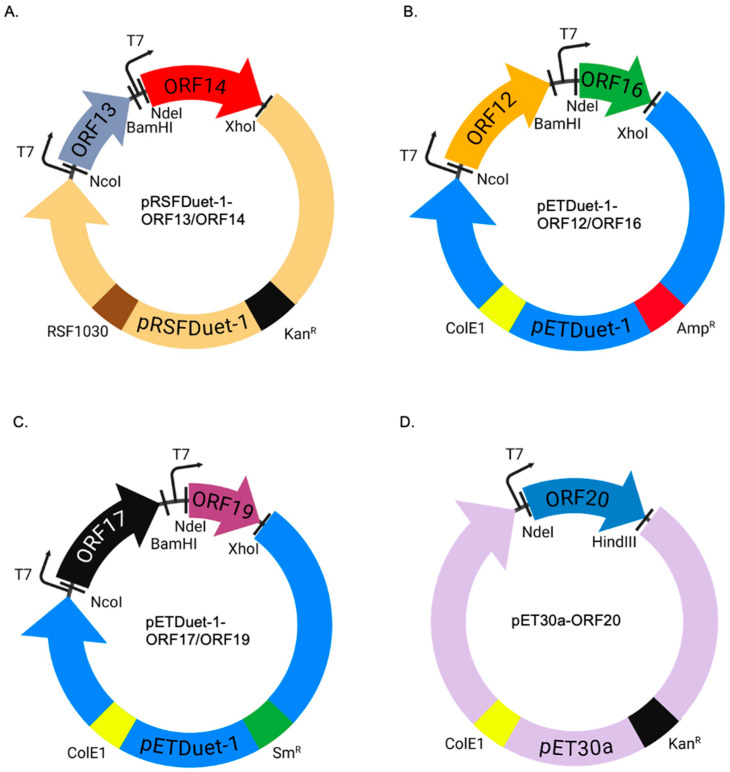
Design of the expression of ORFs in *E. coli* bacteria. (**A**) ORF13 and ORF14 were cloned into pRSFDuet-1 using multiple cloning sites (MCS) I and II, respectively, using the indicated restriction sites. (**B**) ORF12 and ORF16 were cloned into pETDuet-1 using multiple cloning sites (MCS) I and II, respectively, using the indicated restriction sites. (**C**) ORF17 and ORF19 were cloned into pETDuet-1 using MCS I and II, respectively, using the indicated restriction sites. (**D**) ORF20 was cloned into pET30a using multiple cloning sites MCS II using the indicated restriction sites. T7: T7 promoter. RSF1030 and ColE1: origins of replication. Kan^R^, Amp^R^, and Sm^R^: Kanamycin, Ampicillin and streptomycin resistance genes, respectively.

Seven proteins (ORF12, ORF13, ORF14, ORF16, ORF17, ORF19, and ORF20) in the genome of thermophilic bacteriophage ΦIN93 are considered to be structural proteins based on sequence identity with those in the genome of thermophilic bacteriophage P23-77 (Table 1) [2,3]. ORF13 and ORF14 are considered to be capsid proteins, while ORF12, ORF16, ORF17, ORF19, and ORF20 are considered to be membrane-associated proteins. While the expression/co-expression of the two putative capsid proteins have been obtained in the past (and were shown not to form VLPs) [4], the expression of the membrane-associated proteins has never been attempted. Here, we assessed, for the first time, the expression/co-expression of ORF12, ORF16, ORF17, ORF19, and ORF20 (including ORF13 and ORF14) using different expression systems/vectors to see if they can assemble to form VLPs; the proteins in polycistronic constructs (Figure 9A,B) were expressed in thermophilic bacteria, *HB27:nar* strain, while those in monocistronic constructs were expressed in *E. coli* (Figure 10A–D). Expression in *HB27:nar* strain was done at 70 °C to mimic the natural host and the temperature at which the virus normally replicates. The GC content of ΦIN93 virus is very high (66%) and capsid proteins from a related virus, P23-77, are thermostable at temperatures >80 °C [3,7,8]. Thus, we did not expect expression at 70 °C, the proteins in this study, to affect the integrity of the proteins. The polycistronic constructs, expressed here in *HB27:nar,* were designed to mimic the organization of the ORFs in the genome of the virus and expressions were done in host cell/growth conditions that mimicked the host bacteria, *Thermus thermophilus*. Four of these proteins could be co-expressed in a strain of this bacterium, HB27:nar. ORF13 and ORF14 were expressed from pMKE2-ORF13/ORF14 construct while ORF12 and ORF17 were expressed from pMKE2-ORF12/ORF16/ORF17/ORF19/ORF20 construct (Figure 2 and Figure 3). As mentioned in the results, a band of the same size (~14.6 KDa) as ORF12 was also observed in uninduced lysates (Figure 2C and Figure 3C); we believe this band in uninduced sample may be samples that spilled over during loading of samples from induced lysates in neighboring well. This speculation is supported by the fact that the intensity of the band (in uninduced sample) is lower than that in induced sample. Alternatively, this could be leaky expression of the construct, which is supported by the fact that lysates from *HB27:nar* without the expression vector did not have this band. In addition to the detection of ORF12 in induced samples, there was a band at ~25 KDa, which we think is ORF17, even though the size was bigger than the predicted molecular weight of ORF17 (23.3 KDa); we think this band is ORF17 because ORF17 is the only protein (of the five proteins expressed from this polycistronic construct) that has a molecular weight between 20 KDa and 30 KDa. This size for ORF17 is further supported by a previous study that analyzed, on an SDS PAGE gel, proteins from ΦIN93 virion; in this study, the molecular weight estimated by SDS PAGE gel for ORF17 was 26 KDa [16]. Thus, we are confident that the band at ~25 KDa is ORF17. We suspect that the higher molecular weight on the SDS PAGE may be due to the number of prolines in the protein. Studies have shown that high proline levels on protein can slow the migration of the protein on an SDS PAGE gel [24,25,26]. Among the five membrane-associated proteins expressed, ORF17, ORF19, and ORF20 proteins are composed of more than 10% proline residues. ORF17 has a proline ratio of 11%, ORF19 has a proline ratio of 12%, while ORF20 proline’s ratio is 13%; ORF19 and ORF20 proteins migrated at ~14.6 KDa and 11-12 KDa, respectively, as opposed to their predicted sizes of 10.3 KDa and 7.7 KDa (Figure 5C,D and Figure 6A,C).

For the monocistronic constructs expressed in *E. coli*, we chose different vectors (pETDuet-1, pRSFDuet-1, pCDFDuet-1, and pET30a) for co-expression. These vectors were chosen because they have different origins of replication and antibiotic-resistance; both features are necessary for co-existence of two different plasmids in the same cell. All five membrane-associated proteins could be expressed in pairs (ORF12 with ORF16 in pETDuet-1 with amp^R^, ORF17 with ORF19 in pETDuet-1 with Sm^R^, and ORF20 with Kan^R^; Figure 5A–D); same with putative capsid proteins (ORF13 with ORF14 in pRSFDuet-1 with Kan^R^). Although ORF17 and ORF19 could be expressed in pETDuet-1 (Sm^R^) vector, the same proteins could not be expressed in pRSFDuet-1. It is unlikely that the transcription and translation of these two proteins was an issue in this vector given the fact that the promoter (T7) and ribosome binding site of pRSFDuet-1 vector is the same as in pETDuet-1 where the proteins could be expressed. Also, we do not think lack of expression was only due to pRSFDuet-1 vector; this is because ORF13 with ORF14 could be successfully expressed from this vector (Figure 5A). We suspect that lack of expression (that was visible) of ORF17 and ORF19 in pRSFDuet-1 vector could be due to a combination of the vector and ORF17 & ORF19; i.e., the copy number of pRSFDuet-1 vector with ORF17 and ORF19 insertions. Among all the plasmids used in this study, pRSFDuet-1 has the highest copy number (>100), while pETDuet-1 has a copy number of ~40. pCDFDuet-1, on the other hand, has a copy number of >20–40. While a high copy number of a vector in cells is normally associated with increased proteins expression, it can also be associated with metabolic burden on the host cells with little or no protein expression [27,28,29,30]. Thus, we suspect that ORF17 and ORF19 together with pRSFDuet-1 vector (a high copy number plasmid) may be putting a metabolic burden in bacteria that may be affecting the replication/stability of the plasmid and consequently the expression of ORF17 and ORF19.

Following successful expression of all five membrane-associated proteins (ORF12, ORF16, ORF17, ORF19, and ORF20) in the vectors above, we compared their expression levels in four different strains of *E. coli* (C41, Origami 2, Rosetta 2, and BL21 Star). C41 cells promote the expression of toxic or membrane associated proteins, while Origami 2 cells promote the formation of disulfide bonds. BL21 Star is a strain of BL21 cells with a mutation in rne131 gene that codes for RNase E. This mutation enhances RNA stability and consequently high levels of protein expression. Rosetta 2, on the other hand, is a strain of BL21 cells with additional copies of genes that code for rare tRNA; this promotes the expression of heterologous proteins with rare codons in bacteria [31]. Among the four strains of *E. coli* tested, expression levels were better in BL21 Star followed by Rosetta 2 (Figure 5A and Figure 6A). These data suggest that the expression levels of these ORFs may be dependent on the stability of their mRNA (based on high expression levels in BL21 Star), as well as the presence of rare tRNAs (based on results following expression in Rosetta 2 cells). The latter also suggest that codon optimization alone may not be enough to co-express these ORFs at high levels in *E. coli* given the fact the ORFs were codon optimized for *E. coli* expression, but they could only be expressed at suboptimal levels in C41 cells and Origami 2 cells. While the co-expression of ORF12, ORF13, ORF14, ORF16, and ORF17 in BL21 Star was evident by Western blot, that of ORF19 was difficult to confirm for the following reason: The predicted size for ORF19 (based on amino acid sequence) is 10.3 KDa; however, it migrated on SDS PAGE gel close to the size of ORF 12 (14.6 KDa; Figure 5C and Figure 6A,C). Thus, it is likely that ORF19 was expressed, but it could not be seen due to the fact that it co-migrated at the same position as ORF12. As mentioned above, ORF19 has a proline ratio of 12%; this could have affected the migration and the size of the protein on an SDS PAGE gel. Analysis of co-expressed proteins by TEM showed oval structures (Supplementary Materials, Figure S1B) that are similar to published structure of a related thermophilic bacteriophage, P23-77 [8]. Nevertheless, the sizes (~100 nm) of the oval structures were less than the size of authentic ΦIN93 virus (130). Thus, future studies are needed to confirm if these oval structures are VLPs or not.

## 4. Materials and Methods

### 4.1. Cloning of Coat Proteins and Membrane-Associated Proteins in Expression Vectors

As mentioned above, we had previously co-expressed ORF13 and ORF14 using C41 *E. coli* strain in a prior study [4]. To mimic the natural environment and conditions where these coat proteins and membrane-associated proteins are normally expressed, we decided to express them in a format (genomic organization) and at conditions that mimic the host of ΦIN93 virus (i.e., in a thermophilic bacterium). DNA sequences corresponding to the open reading frames of coat proteins (ORF13 and ORF14), including the 9-base pair sequence (5′ GGAGGGACT 3′), that separate the two coat proteins were synthesized by Epoch Life Sciences (Figure 9A). The nine nucleotides included part of a native Shine–Dalgarno sequence present in the genome ΦIN93, which is upstream of ORF14. The DNA fragment was amplified by PCR, digested and cloned into pMKE2 vector (a gift from Dr. Jose Berenguer, Universidad Autónoma de Madrid); cloning was performed downstream of a respiratory nitrate reductase promoter (Pnar) using NcoI and EcoRI sites. pMKE2 vector is a thermophilic vector that allows the expression of foreign proteins (from Pnar) in thermophilic bacteria [32,33]. To express ORF12, ORF16, ORF17, ORF19, and ORF20, a polycistronic construct that has DNA sequences of the five open reading frames, separated by up to 19-base pair sequence (with native Shine–Dalgarno sequence in the genome of ΦIN93 inclusive) was also synthesized and cloned to pMKE2 vector (Figure 9B) as described above. All constructs were sequenced across cloning junctions to confirm the authenticity of the genes.

For the expression of the proteins (ORF12, ORF13, ORF14, ORF16, ORF17, ORF19, and ORF20) in *E. coli*, genes that code for the proteins were codon-optimized for *E. coli* expression. The codon-optimized genes were synthesized and cloned into bacterial expression vectors (by Epoch Life Sciences) for co-expression as follows: ORF13 and ORF14 were cloned to multiple cloning sites (MCS)I and MCSII, respectively, in pRSFDuet-1; ORF12 and ORF16 or ORF17 and ORF19 were cloned into pETDuet-1 vector while ORF20 was cloned into pET30a vector (Figure 10A–D).

### 4.2. Co-Expression of Coat Proteins and Membrane-Associated Proteins in a Thermophilic Bacterium (HB27:nar) and in E. coli

To check if the coat proteins and membrane-associated proteins could be expressed, the bicistronic and polycistronic vectors (pMKE2) were used to transform a thermophilic bacterium, *Thermus thermophilus HB27:nar* (also a gift from Dr. Jose Berenguer). As mentioned above, *HB27:nar* is *a* derivative of HB27 strain that carries a respiratory nitrate reductase (nar) operon, which allows the bacteria to also grow anaerobically [22]. Transformation was performed as follows: *HB27:nar* was grown overnight at 70 °C in Terrific broth medium (TB medium: 8 g peptone, 4 g yeast extract, 3 g NaCl, pH 7.5). Overnight cultures were diluted in 1:50 fresh TB medium and grown at 200 rpm at 70 °C until an OD_600_ of 0.4. Eight hundred micro liters of the cells were transformed with 300 ng of the plasmids (pMKE2 with ORF13 and ORF14 insertions or pMKE2 with ORF12, ORF16, ORF17, ORF19 and ORF20 insertions) and cultured at 200 rpm at 70 °C for an additional 4 h. The transformants were concentrated to 100 µL (by centrifugation at 3000 rpm) and then plated on 3% Terrific broth agar plates containing 30 µg kanamycin; the plates were incubated at 70 °C for 2 to 3 days. To screen for protein expression, colonies were picked up from the agar plates and were individually inoculated into 5 mL TB medium containing 30 µg kanamycin. The mixture was grown at 70 °C (shaking at 200 rpm) until an OD_600_ of 0.4. Protein expression was induced by adding 40 mM potassium nitrate (KNO_3_) and the cells were anaerobically incubated (without shaking) at same temperature for 4 h. To check for protein expression, cultures were pelleted, lysed, and the lysate/supernatant were run on SDS-PAGE followed by Western blotting. Cultures that showed protein expression (of expected sizes) were used to isolate plasmids for co-expression of the two plasmids (pMKE2-ORF13/ORF14 and pMKE2-ORF12/ORF16/ORF17/ORF19/ORF20). Co-expression of the proteins from two plasmids was performed by mixing the two plasmids in equal concentrations (300 ng) and transforming *HB27:nar* bacteria as described above using the same antibiotic concentration. Protein expression and induction was performed as described above.

To co-express the proteins in *E coli*, constructs (Figure 10A–D) expressing ORF12 and ORF16, OFR13 and ORF14, ORF17 and ORF19, and ORF20 were separately transformed into 2-4 strains of *E. coli* [C41(DE3), Rosetta 2(DE3)pLysS, Origami 2(DE3)pLysS cells, and BL21 Star(DE3)]. Protein expression and levels of expression were screened as previously described [34]. Following the confirmation of protein expression by SDS PAGE and/or Western blots, all four expression vectors were used to co-transform BL21 Star cells.

### 4.3. Ultracentrifugation of Co-Expressed Proteins to Assess the Formation of VLPs

For expression in *thermophilic bacteria* (HB27:nar) and *E. coli* (BL21 Star), lysates of bacteria from co-expression of ORF13/ORF14 and ORF12/ORF16/ORF17/ORF19/ORF20 were spun at 10,000 rpm for 10 min. Their supernatants were run on a cesium chloride density gradient (1.25 g/mL and 1.35 g/mL). The samples were centrifuged at 20,000 rpm for 16 h (4 °C) and different bands were collected for SDS PAGE, Western blots, and TEM analysis.

### 4.4. Western Blot on Bacterial Lysates and on Bands from Ultracentrifugation

Lysates from bacteria transformed/co-transformed with pMKE2-ORF13/ORF14 and pMKE2-ORF12/ORF16/ORF17/ORF19/ORF20, as well as lysates from bacteria transformed/co-transformed with plasmids pRSFDuet-1, pETDuet-1, and pET30a were resolved on SDS PAGE gels and transferred onto polyvinylidene difluoride membranes. Bands from ultracentrifugation of supernatants derived from the lysates were also resolved and transferred to the membranes. The membranes were blocked and 1:250–1:2000 dilution of ORF13 and ORF14 mixture, ORF12-ORF16-ORF17-ORF19-ORF20 recombinant protein polyclonal sera (generated in our lab; Supplementary Materials, Figures S2–S5) were added and incubated for 1 h. Horseradish peroxidase-conjugated goat anti-mouse IgG antibodies (1:10,000 dilution) were added to the membranes and they were developed using SuperSignal West Pico (Luminol/Enhancer and stable peroxide) solutions.

### 4.5. Transmission Electron Microscopy (TEM)

To assess the potential of coat proteins to assemble into VLPs, TEM was conducted using samples derived from bands from ultracentrifugation which contain (by Western blot) coat proteins and membrane-associated proteins. The samples were loaded onto glow-discharged carbon grids for 2 min. The grids were stained with 2% uranyl acetate for 2 min. Samples were visualized using a Hitachi H-7650 Transmission Electron Microscope.

## 5. Conclusions

The co-expression of ORF12, ORF13, ORF14, ORF16, and ORF17 in this study did not lead to the formation of VLPs, which was a little disappointing. Irrespective of this, this study has significant information that can help advance the field to develop thermostable VLPs with broader applications. For example, our study is the first to evaluate the expression of membrane-associated proteins (and putative capsid proteins) from bacteriophage ΦIN93 in a thermophilic bacterium and in *E. coli*. Additionally, this is the first study to co-express more than three types of heterologous proteins in a thermophilic bacterium using the same type of plasmid. Furthermore, ~5 of these proteins (Figure 7A) could be co-expressed from 3 plasmids (two of which had the same origin of replications but different antibiotic-resistance genes) in *E. coli*. Moreover, we believe co-expressing these proteins together from different plasmids is a good approach to assess which of them may be required to form VLPs. It is generally believed that plasmids with the same origins of replication cannot be expressed in the same bacteria due to incompatibility in origins of replication. Here, we demonstrated that two pETDuet-1 plasmids (both with CoE1 origin of replication but with ampicillin and streptomycin resistance genes) and pRSFDuet-1 plasmid (with RSF1030, kanamycin resistance) could be co-expressed together. Overall, the expression levels of the ORFs in *E. coli* BL21 Star were better than those in other *E. coli* strains tested (Rosetta 2, C41, and Origami 2), which were, in turn, better than expression levels in thermophilic bacteria, *HB27:nar*. Thus, BL21 Star is a better system to express high levels of ORFs from ΦIN93 than the thermophilic bacteria, *HB27:nar*.

## Figures and Tables

**Table 1:** Properties of ΦIN93 structural proteins in comparison to P23-77.

ΦIN93 Protein	Predicted Molecular Weight (KDa) *	Predicted Function	Homolog in P23-77 (% Homology)
ORF12	14.6	Membrane-associate protein	VP15 (~83%)
ORF13	19.3	capsid protein	VP16 (~80%)
ORF14	32.0	capsid protein	VP17 (73%)
ORF16	8.9	Membrane-associate protein	VP19 (~68%)
ORF17	23.3	Membrane-associate protein	VP20 (~69%)
ORF19	10.3	Membrane-associate protein	VP22 (~51%)
ORF20	7.7	Membrane-associate protein	VP23 (~56%)

* Predicted weight based on amino acids.

## Data Availability

Additional data contained within this article is in Supplementary Materials.

## Supplementary Materials

The supporting information can be downloaded at: https://www.mdpi.com/article/10.3390/ijms26115201/s1.
