# Supplementary material for: Design, Co-Expression, and Evaluation for Assembly of the Structural Proteins from Thermophilic Bacteriophage ΦIN93
Source: Int J Mol Sci. 2025 May 28;26(11):5201. doi: 10.3390/ijms26115201 (PMC12154516; doi:10.3390/ijms26115201)
Supplement: Supplementary file 1 [file ijms-26-05201-s001.zip › ijms-3598645-supplementary.pdf]

Supplementary Information

TEM analysis of layer 4 from density gradient ultracentrifugation showed oval structures with an average size of ~62-100 nm (Supplemental Figure S1)

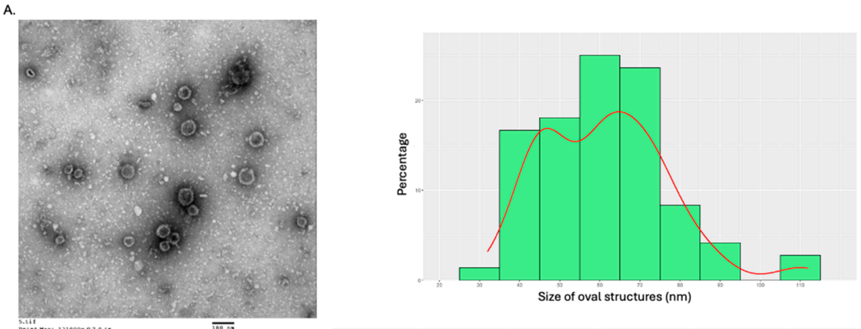

Mean	Median	Min	Max	Std Dev	Std Error
61.34	61.77	32.03	111.83	15.81	1.86

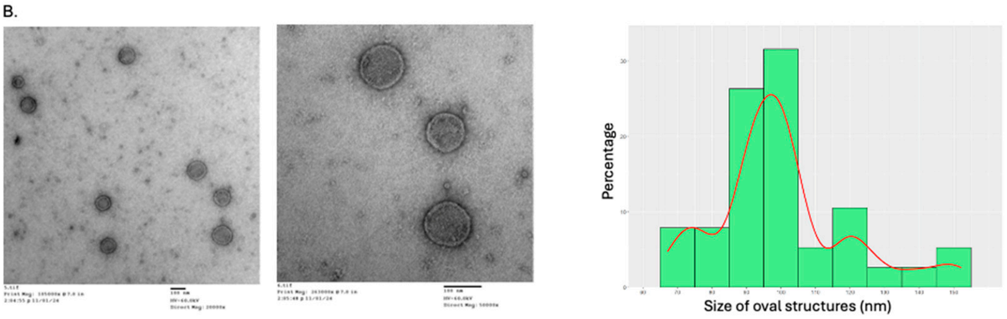

mean	median	min	max	Std Dev	Std Error
100.25	98.57	67.23	152.09	19.44	3.15

Supplemental Figure S1: TEM analyses. Samples from layer 4 of ultracentrifugation of supernatants from proteins expressed in HB27:nar (A) or in BL21 Star (B) were loaded onto glow-discharged carbon grids for 2 minutes, stained with 2% uranyl acetate and visualized using a transmission electron microscope. Graph on the right is a size distribution of the oval structures from TEM.

Supplemental Materials and Methods

Expression and purification of ORF13, ORF14 and ORF12-ORF16-ORF17-ORF19-ORF20 recombinant protein

Genes that code for ORF12, ORF16, ORF17, ORF19 and ORF20 were codon-optimized for bacterial expression and synthesized as one ORF fragment without start and stop codons between each of the ORFs; three glycine residues were included between each

of the ORFs to enhance flexibility of individual proteins (Supplemental Figure S2A). Six-histidine residues were added to the C-terminus of the gene fragment for affinity purification. The recombinant protein was cloned into pET28a vector using NcoI/BamHI sites. The recombinant protein was expressed and purified as previously described [13] with a few modifications. Bacterial pellet was first lysed using BugBuster protein extraction reagent (Novagen), centrifuged and the pellet was responded in 2 M urea (supplemented with 20 mM NaH_2PO_4, 20 mM Na_2HPO_4, 50 mM imidazole, 400 mM NaCl, 10% tween 20, 10 mM beta-mercaptoethanol). The mixture was sonicated, spun, and the supernatant added to Ni-NTA beads. The beads were washed 5X with 2 M urea buffer. Recombinant protein was eluted from the column using Ni-NTA elution buffer (Sigma-Millipore) and the protein was refolded by dialysis as follows. The protein was dialyzed overnight in refolding buffer 1 (0.5 M urea, 20 mM NaH_2PO_4, 5 mM reduced glutathione, 0.5 mM oxidized glutathione, 0.5 M arginine, 300 mM NaCl, 10% glycerol, pH 7.5) followed by dialysis for 4 hours in refolding buffer 2 (20 mM NaH_2PO4, 150 mM NaCl, 10% glycerol, pH 7.5).

The genes that code for ORF13 and ORF14 were codon-optimized for E. coli expression; the genes were cloned into PET15b and pET28a vectors (respectively; Supplemental Figure S2B) and expressed in C41 cells. Protein expression was induced with 0.5 mM IPTG. Expressed proteins were purified by size exclusion chromatography (Sepharose CL-4B beads) followed by anion exchange chromatography.

A.

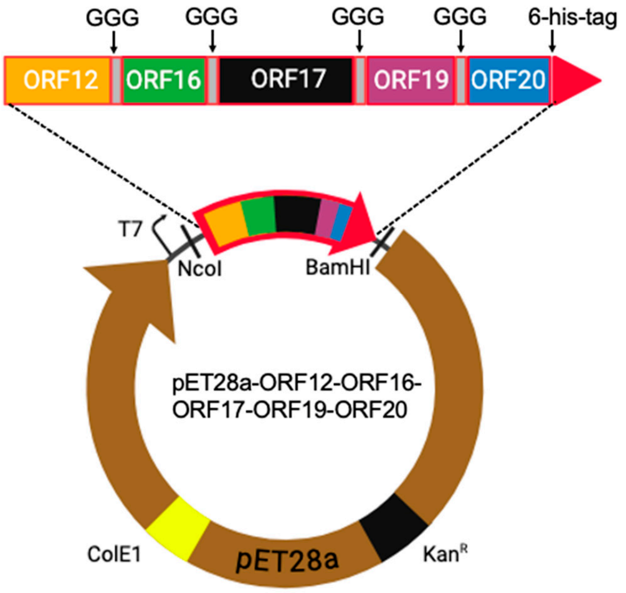

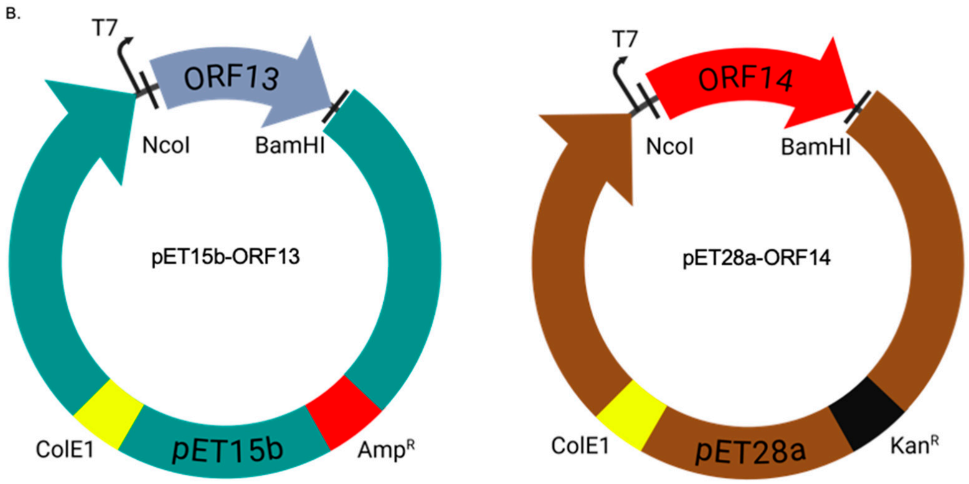

Supplemental Figure S2. Design of the expression of ORFs in *E. coli* bacteria. A) ORF12-ORF16-ORF17-ORF19-ORF20 recombinant protein was cloned into pET28a. B) ORF13 and ORF14 were cloned into pET15b and pET28a, respectively. All cloning was done using the indicated restriction sites. T7: T7 promoter. ColE1: origins of replication. Kan^R and Amp^R are Kanamycin and Ampicillin and resistance genes, respectively.

Generation of polyclonal antibodies against ORF13, ORF14, and ORF12-ORF16-ORF17-ORF19-ORF20 recombinant protein

Animal work was approved by Texas Tech University Health Sciences Center IACUC (Institutional Animal Care and Use Committee). To generate polyclonal antibodies, 10 µg each of purified ORF13, ORF14 or recombinant protein (ORF12-ORF16-ORF17-ORF19-ORF20) were used to subcutaneously immunize Balb/c mice; all immunizations were done with alum adjuvant and mice were immunized 3 times at two-week intervals. Two weeks after the last immunizations, whole blood was collected from mice and antibody titers (total IgG) in sera were determined using the above proteins as target antigens.

Supplementary Results

The expression of ORF12-ORF16-ORF17-ORF19-ORF20 recombinant protein in C41 cells was not visible after lysates of the bacterial were run on SDS PAGE gel and stained

with Coomassie blue dye (Supplemental Figure S3A). However, when the protein was expressed in Rosetta cells, low levels of expression was observed. A faint band at ~65.8 KDa (the expected size of the recombinant protein) was observed in induced sample but not in the uninduced sample (Supplemental Figure S3A). The recombinant protein was purified to homogeneity (Supplemental Figure S3B). ORF13 and ORF14 were successfully expressed at high levels and were purified from bacterial lysates (Supplemental Figure S4A,B).

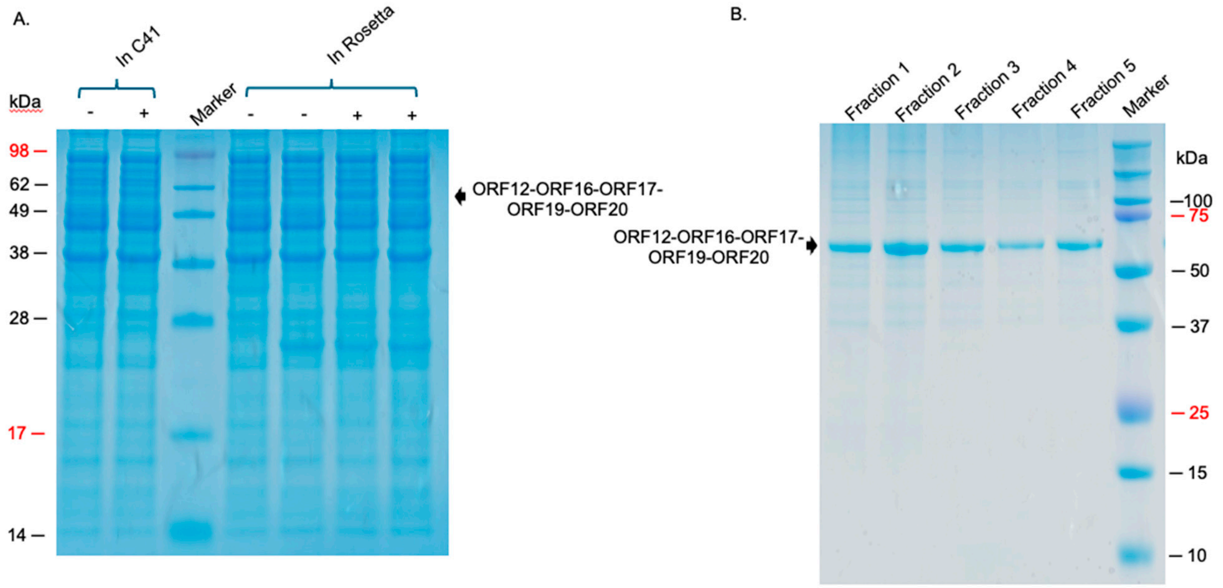

Supplemental Figure S3: Expression and purification of ORF12-ORF16-ORF17-ORF19-ORF20 recombinant protein. A) The 5 ORFs, codon-optimized for E. coli expression, were cloned with a 6-histidine tag into pET28a vector and expressed in C41 cells or Rosetta cells. C41 or Rosetta cells transformed with the vector were induced with 0.5 mM IPTG. Cells were grown for 4 additional hours, lysed and cell lysates were run on a 10% SDS PAGE gel. B) Rosetta cells expressing the proteins in (A) were lysed with 2M urea and the protein was purified on a nickel NTA column. Fractions were collected and run on a 10% SDS PAGE gel followed by staining with Coomassie blue dye.

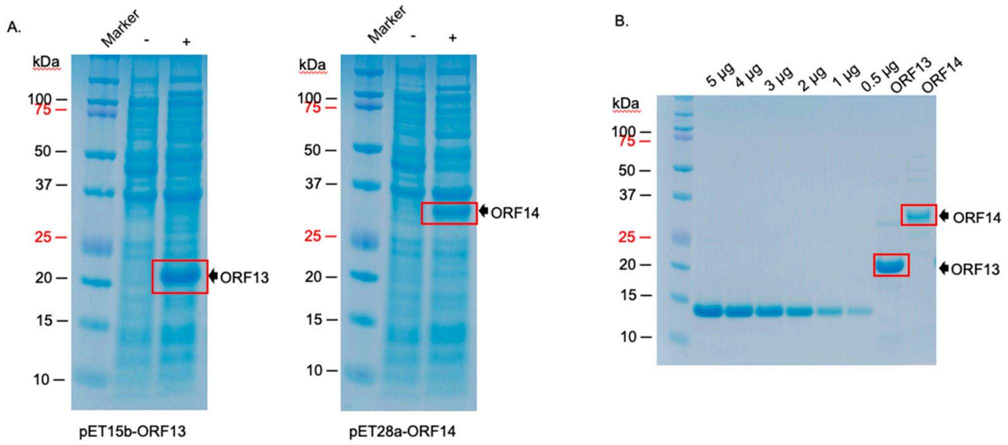

Supplemental Figure S4: Expression and purification of ORF13 and ORF14 proteins. A) The genes for ORF13 and ORF14, codon-optimized for E. coli expression, were cloned into PET15b and pET28a vectors (respectively) and expressed in C41 cells. C41 cells transformed with the vector were induced with 0.5 mM IPTG. Cells were grown for 4 additional hours, lysed and cell lysates were run on a 10% SDS PAGE gel. B) C41 cells expressing the proteins in (A) were lysed with Bugburster buffer and proteins were purified by size exclusion and anion exchange chromatography.

Mice immunized with the purified ORF13, ORF14, and ORF12-ORF16-ORF17-ORF19-ORF20 recombinant protein elicited very high titer IgG antibody response (geometric mean >10^5) compared to control naïve sera (Supplemental Figure S5A–C).

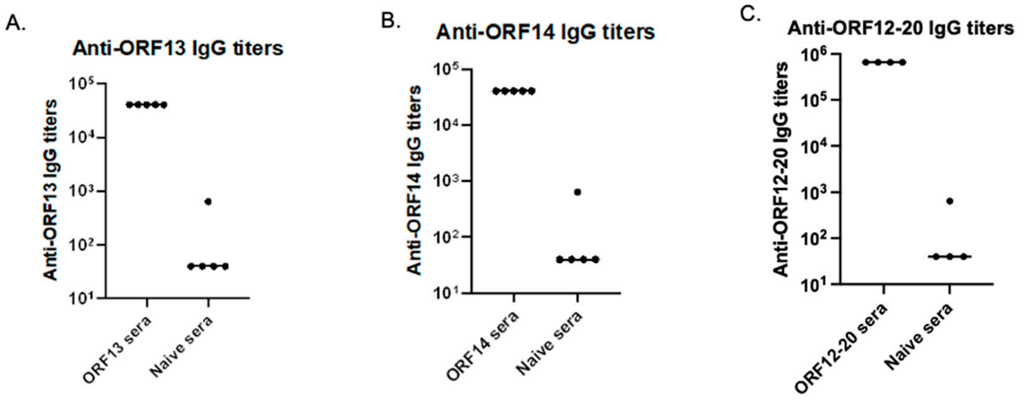

Supplemental Figure S5: Immunogenicity of ORF13, ORF14, and ORF12-ORF16-ORF17-ORF19-ORF20 recombinant protein. Balb/c mice were immunized thrice with A) ORF13, B) ORF14, and C) ORF12-ORF16-ORF17-ORF19-ORF20 protein. Whole blood

was collected from mice and IgG titers were determined by ELISAs using the respective proteins as target antigens.
